# The use of high-affinity polyhistidine binders as masking probes for the selection of an NDM-1 specific aptamer

**DOI:** 10.1038/s41598-022-12062-2

**Published:** 2022-05-13

**Authors:** Wiebke Sabrowski, Nico Dreymann, Anja Möller, Denise Czepluch, Patricia P. Albani, Dimitrios Theodoridis, Marcus M. Menger

**Affiliations:** 1 Fraunhofer Institute for Cell Therapy and Immunology, Branch Bioanalytics and Bioprocesses (IZI-BB), Am Mühlenberg 13, 14476 Potsdam, Germany; 2 Institute of Chemistry and Biochemistry – Biochemistry, Freie Universität Berlin, Takustr. 6, 14195 Berlin, Germany; 3 Institute of Biochemistry and Biology, University of Potsdam, Karl-Liebknecht Strasse 24-25, 14476 Potsdam-Golm, Germany; 4 nal von minden GmbH, Robert-Bosch-Breite 23, 37079 Göttingen, Germany

**Keywords:** DNA, Antimicrobial resistance, Biochemical assays

## Abstract

The emergence of carbapenemase-producing multi-drug resistant Enterobacteriaceae poses a dramatic, world-wide health risk. Limited treatment options and a lack of easy-to-use methods for the detection of infections with multi-drug resistant bacteria leave the health-care system with a fast-growing challenge. Aptamers are single stranded DNA or RNA molecules that bind to their targets with high affinity and specificity and can therefore serve as outstanding detection probes. However, an effective aptamer selection process is often hampered by non-specific binding. When selections are carried out against recombinant proteins, purification tags (e.g. polyhistidine) serve as attractive side targets, which may impede protein target binding. In this study, aptamer selection was carried out against N-terminally hexa-histidine tagged New Delhi metallo-ß-lactamase 1. After 14 selection rounds binding to polyhistidine was detected rather than to New Delhi metallo-ß-lactamase 1. Hence, the selection strategy was changed. As one aptamer candidate showed remarkable binding affinity to polyhistidine, it was used as a masking probe and selection was restarted from selection round 10. Finally, after three consecutive selection rounds, an aptamer with specific binding properties to New Delhi metallo-ß-lactamase 1 was identified. This aptamer may serve as a much-needed detection probe for New Delhi metallo-ß-lactamase 1 expressing Enterobacteriaceae.

## Introduction

Antibiotics are the most important line in the defense against bacterial infections. Since their discovery more than 70 years ago, antibiotics have enabled the treatment of previously life-threatening conditions^1^. However, overuse and misuse of these medications resulted in a rapid emergence of resistant bacteria^2^. In 2019, the World Health Organization (WHO) declared antimicrobial resistances as one of the top ten health threats worldwide, ranking carbapenem resistance as the most crucial concern^3^. Carbapenem antibiotics are usually considered the most effective drugs to fight infections with multi-drug resistant bacteria and therefore serve as agents of last resort^4^. Yet, carbapenem-resistant Enterobacteriaceae (CRE) and other bacterial families are on the rise leaving the health care system with very little treatment options^5^. Carbapenem resistance in CRE is mainly mediated through the expression of carbapenemases. Carbapenemases are a specialized class of ß-lactamases that harbor the ability to hydrolyze carbapenem antibiotics^6^. ß-lactamases can be classified by the Ambler classification, dividing them into for subgroups (A-D) owing to their amino acid sequence. Classes A, C and D are serine ß-lactamases, while members of class B require a bivalent metal ion for activity and are therefore described as metallo-ß-lactamases (MBLs)^7^. Prominent carbapenemases belonging to the group of serine ß-lactamases are for example Klebsiella Pneumoniae Carbapenemases (KPCs) and Oxacillinases (OXAs). KPCs are class A ß-lactamases while OXAs are members of the group of class D ß-lactamases. Another highly prevalent carbapenemase, belonging to class B of ß-lactamases, is New Delhi metallo-ß-lactamase 1 (NDM-1).

NDM-1 is a carbapenemase firstly discovered in India which has spread across the globe. The genetic element encoding NDM-1 is located on a highly transmissible plasmid allowing rapid horizontal gene transfer^8^. Even though NDM-1 belongs to the family of class B ß-lactamases, it shares very little identity with other MBLs. Verona-Integron-Metallo-ß-lactamases (VIM1/2) are the most closely related MBLs with only 32.4% consensus^9^. NDM-1 can hydrolyze all ß-lactam antibiotics except for Aztreonam. In contrast to other carbapenemases, NDM-1 is not susceptible to the ß-lactamase inhibitor Avibactam^10^. Hence, treatment options are very limited. Therefore, fast, affordable and comprehensive detection of NDM-1-harboring bacteria is important for both limitation of further spread of carbapenem resistant bacteria and for effective treatment. However, detection methods used to date exhibit certain limitations, such as time-consuming workflows, the need for well-trained personal or the inability to discriminate between different carbapenemase types^11^. Here, a possible solution is the development of detection systems based on highly specific and robust aptamers.

Aptamers are short single-stranded (ss) nucleic acids (ssDNA or RNA) molecules with the ability to bind their target molecule with high specificity and selectivity. Binding is mediated via their three-dimensional structure^12^. Aptamer development is based on an iterative in vitro selection process called SELEX (Systematic Evolution of Ligands by Exponential enrichment) including repeated rounds of DNA-target incubation^13,14^. Aptamers display a range of desirable features, such as high stability over a great range of conditions, regenerative target binding, and chemical synthesis that is free of batch-to-batch variations. Chemical synthesis is low in production costs and allows a broad range of facile chemical modification^15^. Therefore, aptamers serve as excellent detection probes for incorporation into analytical devices like biosensors^16^.

However, the selection of aptamers harbors certain pitfalls. Enrichment of non-specific binders happens frequently throughout the in vitro selection process. Especially, when selecting against recombinant proteins, target purification often requires the incorporation of affinity tags. Furthermore, separation of binding sequences from non-binding sequences regularly necessitates target immobilization. Both, affinity tags as well as the immobilization matrix may serve as additional aptamer binding epitopes^17^. Negative selections with other proteins harboring the same affinity tag as well as the immobilization matrix alone can be used to reduce the enrichment of non-specific binders^18^. However, negative selections may not always be sufficient to eliminate non-specific binders completely.

In this study, we describe how selection against N-terminally hexa-histidine tagged New Delhi metallo-ß-lactamase 1 (HIS-NDM-1) initially resulted in polyhistidine tag (HIS-tag) binding. The identified, HIS-tag binding DNA aptamer and its truncated derivate were found to bind to polyhistidine tagged proteins with up to picomolar affinity. The aptamers truncated derivate NDM1-H14-01-FR was further utilized to reverse HIS-tag binding and facilitate specific target binding. Finally, we introduce a DNA aptamer that binds to HIS-NDM-1 but neither to the N-terminally HIS-tagged carbapenemase OXA-23 (HIS-OXA-23) nor to a synthetic hexa-HIS peptide. Scramble control ConSc as well as unrelated control aptamer Con1 did not show binding to HIS-NDM-1, highlighting sequence dependence of the binding event. Other DNA aptamers, targeting either NDM-1 or NDM-1 and VIM-2 simultaneously, were patented in 2012 and 2018^19,20^. Here, affinity to NDM-1 or to NDM-1 and VIM-2 was quantified using surface plasmon resonance spectroscopy (SPR), while specificity and sequence dependence of the binding event were not assessed. Based on the comprehensive characterization of our aptamer by different methods and controls, we believe it can serve as a valuable binding probe for the detection of NDM-1. The development of new detection systems for NDM-1 provides the opportunity to prevent the further spread of carbapenem-resistant bacteria and the mismanagement of infections caused by antibiotic-resistant bacteria.

## Results

### First selection rounds resulted in the selection of HIS-tag binding aptamers

Eleven selection rounds (SR) were performed against HIS-NDM-1 covalently coupled to tosylactivated magnetic beads. Varying numbers of negative selections against beads only were carried out prior to target incubation in every selection round (Supplementary Table S1). Additionally, for SR5.1–7.2, negative selection was performed against N-terminally HIS-tagged KPC-2 (HIS-KPC-2) and for SR8.1–11.1 with both HIS-KPC-2 and HIS-OXA-23. To monitor enrichment of matrix binders, control selections with tosylactivated beads only were carried out frequently throughout the selection process (SRX.X_Control). After SR11.1, enriched DNA-pools from SR10.1, 11.1, and 11.1_Control were analyzed utilizing next generation sequencing (NGS) and bioinformatics. Pool enrichment was rather low with 71.4% unique sequences for SR10.1, and 65.3% for SR11.1. Enrichment of control selection 11.1_Control was also low with 75.6% unique sequences. For SR11.1, the most enriched pattern accounted for only 0.4% of total sequences and analysis of the 50 most enriched sequences revealed only little homology. Yet, 9 clones were screened for binding to NDM-1. Clone selection was performed based on two criteria. First, overall enrichment after SR11.1 was considered, and second, clones were selected and synthesized on the condition that the percentage of total sequences was higher for SR11.1 than for SR11.1_Control, since stronger enrichment in control selections indicates matrix binding. Selected clones were screened for binding to HIS-NDM-1 using fluorescent dye-linked aptamer assay (FLAA) and SPR. No binding was detected (Supplementary Fig. S1). To lower selection pressure and thereby possibly facilitate target binding, three more selection rounds were conducted in which negative selections were limited to tosylactivated magnetic beads only. After 14 selection rounds, enriched DNA-pools from selection rounds 13.1, 14.1, and 14.1_Control were sequenced by NGS and analyzed bioinformatically. Here, enrichment for target selections was still low with 75.3% unique sequences for SR13.1, and 64.8% for SR 14.1, whereas SR14.1_Control was strongly enriched with only 38.8% unique sequences. However, for SR14.1, the most enriched pattern accounted for 4.72% of total sequences and analysis of the 50 most enriched clones revealed five distinct motives that were present in a high number of sequences. Sequences bearing the same motive were clustered as four sequence families. Representatives from each family were selected and synthesized on the condition that the percentage of total sequences was higher for SR14.1 than for SR14.1_Control.

**Figure 1:**
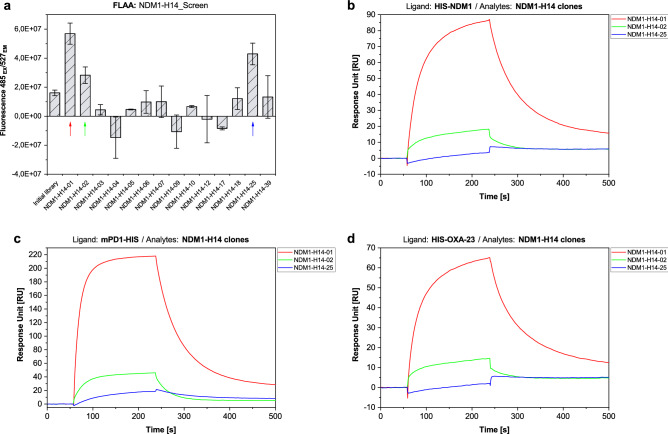
Aptamer candidate screening after SR14.1. HIS-NDM-1 was immobilized on a micro titer plate and incubated with aptamer candidates for FLAA (**a**). For further characterization of promising clones NDM1-H14-01, NDM1-H14-02, and NDM1-H14-25, HIS-NDM1, HIS-OXA-23, and mPD1-HIS were immobilized on an SPR sensor chip and aptamer candidates were injected (**b**–**d**). Binding signals were obtained for clones NDM1-H14-01 and NDM1-H14-02 for all three proteins with strong binding signals for NDM1-H14-01 and weaker binding signals NDM1-H14-02. For NDM1-H14-25, binding was only detected when injected into the mPD1-HIS-channel (**c**). Buffer signals were subtracted. Error bars reflect the range of signals from two different target-coated wells plus the range of two negative control wells in FLAA.

To test binding properties, aptamer candidates were screened for binding to HIS-NDM-1 by FLAA using Greiner FLUOTRAC™ 600 microplates. High binding signals were obtained for the aptamer candidate NDM1-H14-01, clearly exceeding signals obtained from incubation with the initial library used as a negative control. Furthermore, clones NDM1-H14-02 and NDM1-H14-25 also showed signals exceeding that of the initial library. Binding screening for these three aptamer candidates was also performed using SPR. Here, HIS-NDM-1, HIS-OXA-23 (carbapenemase negative control), as well as an unrelated C-terminally HIS-tagged protein—mouse Programmed cell Death protein 1 (mPD1-HIS; HIS-tag negative control), were immobilized as ligands and aptamer candidates were injected as analytes. Surprisingly, binding of clones NDM1-H14-01 and NDM1-H14-02 was detected for all three proteins, while clone NDM1-H14-25 only bound to mPD1. Binding to all three HIS-tagged proteins or to only one, unrelated HIS-tagged protein indicates polyhistidine binding rather than binding to NDM-1 (Fig. 1).

**Figure 2:**
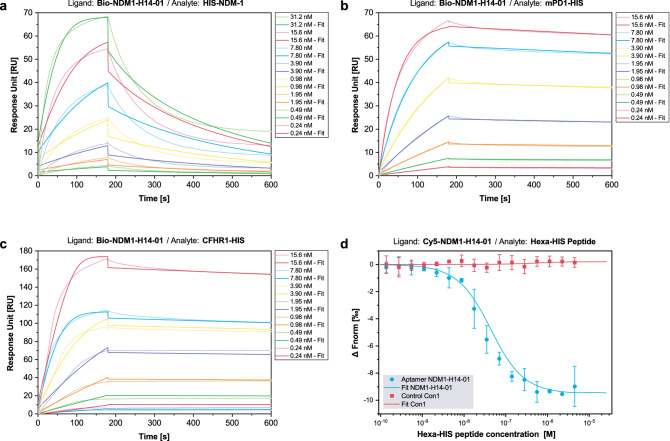
Characterization of NDM1-H14-01 binding to polyhistidine. Binding was analyzed using SPR (**a**–**c**) and MST (**d**). Binding was confirmed for HIS-NDM-1 (**a**), mPD1-HIS (**b**), CFHR1-HIS (**c**), and the hexa-HIS peptide (**d**). K_D_-values ranged from the mid-picomolar to the mid-nanomolar range (Table 1). Error bars reflect the standard deviation from three independent experiments in MST.

As the highest binding signals in both SPR and FLAA were obtained for NDM1-H14-01, it was chosen for further characterization of potential HIS-tag binding. 5′-biotinylated NDM1-H14-01 was immobilized on an SPR sensor chip as a ligand and varying concentrations of HIS-NDM-1, as well as two unrelated C-terminally HIS-tagged proteins, mPD1-HIS and Complement factor H-related protein 1 (CFHR1-HIS), were injected as analytes. Determined dissociation constants (K_D_) varied between 4.49 × 10^−11^ and 4.43 × 10^−9^ M for the three different proteins (Table 1). Hence, highly affine binding to three HIS-tagged proteins was shown. However, to exclude binding to a common protein motive and to demonstrate HIS-tag binding, aptamers were tested for binding to a synthetic, 840 Da hexa-HIS peptide. Due to its low molecular size, hexa-HIS peptide was neither suitable for SPR injection, nor for immobilization-based methods, as immobilization can lead to false negative results. Hence, binding was characterized via immobilization-free Microscale Thermophoresis (MST). Here, 5′-Cy5-labelled NDM1-H14-01, as well as an unrelated control aptamer (Con1) were incubated with varying concentrations of the hexa-HIS peptide. A K_D_ of 2.81 ± 0.39 × 10^−8^ M was calculated for NDM1-H14-01, while Con1 did not show binding. Thus, the highly affine HIS-tag binding indicated by SPR was confirmed by MST (Fig. 2).

**Table 1:** Quantitative characterization of binding parameters of NDM1-H14-01 and NDM1-H14-01-FR using SPR and MST.

Method	Ligand	Analyte	k_a_ (1/Ms)	k_d_ (1/s)	K_D_ (M)	Chi^2^ (RU^2^)	K_D_ confidence (M)
SPR	Bio-NDM1-H14-01	HIS-NDM-1	1.30E+06	5.62E−03	4.34E−09	6.47	–
		mPD1-HIS	1.40E+06	1.38E−04	9.86E−11	0.20	–
		CFHR1-HIS	2.79E+06	1.25E−04	4.49E−11	6.97	–
MST	Cy5-NDM1-H14-01	Hexa-HIS-peptide	–	–	2.81E−08	–	3.90E−09
SPR	Bio-NDM1-H14-01-FR	HIS-NDM-1	1.20E+06	7.11E−03	5.91E−09	9.68	–
		mPD1-HIS	1.02E+06	1.28E−04	1.25E−10	0.20	–
		CFHR1-HIS	4.28E+06	1.22E−04	2.86E−11	9.22	–

### Masking approach to prevent enrichment of HIS-tag binding sequences

To avoid HIS-tag binding, negative selections are commonly used. However, in our initial approach, seven selection rounds with negative selections against either one or two related N-terminally HIS-tagged proteins were not sufficient to remove all HIS-tag binding sequences. After only three consecutive rounds without negative selections, HIS-tag binding sequences were strongly enriched and sequences binding specifically to NDM-1 could not be identified. Therefore, we went back to the ssDNA pool from selection round 10.1 and designed a new selection strategy. The ssDNA pool from selection round 10.1 was chosen as sequencing data did not show enrichment of identified HIS-tag binding sequences.

**Figure 3:**
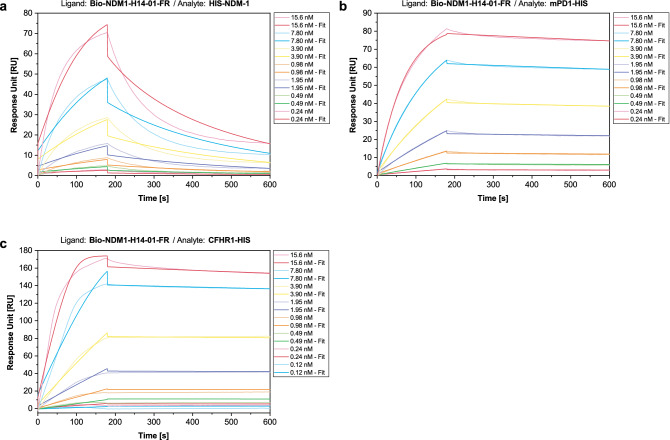
Characterization of NDM1-H14-01-FR binding to polyhistidine. Binding was analyzed using SPR. Binding was confirmed for HIS-NDM-1 (**a**), mPD1-HIS (**b**), and CFHR1-HIS (**c**). K_D_-values were ranging from the mid-picomolar to the mid-nanomolar range (Table 1).

To facilitate NDM-1 binding while preventing HIS-tag binding, a combinatorial approach of negative selection against unrelated HIS-tagged proteins and masking of the HIS-tag was chosen. Here, NDM1-H14-01 was already shown to bind the HIS-tag of various proteins with high affinity and therefore served as an excellent masking aptamer. However, using aptamers from the same selection bears a risk, as both the ssDNA pool and the masking aptamer have the same primer binding sites. PCR amplification after the selection step may lead to high rates of HIS-tag binder amplification and bears the risk of losing specific NDM-1 binders. Hence, to prevent distortion of the selection process by enrichment of NDM1-H14-01 copies, a truncated derivative of the aptamer, lacking both primer binding sites, was designed (NDM1-H14-01-FR). To assess whether HIS-tag binding was retained, SPR-based kinetic measurements were performed as previously described for the full-length aptamer. Binding to all three proteins was preserved and binding was even more affine with binding constants of 5.91 × 10^−9^ M for HIS-NDM-1, 1.25 × 10^−10^ M for mPD1-HIS, and 2.86 × 10^−11^ M for CHFR1-HIS (Fig. 3). K_D_-values and kinetic parameters of these HIS-tag binding aptamers that showed strongest binding parameters are summarized in Table 1. As highly affine binding was retained, NDM1-H14-01-FR was used to mask the HIS-tag of HIS-NDM-1 in further selection rounds.

### Selection against HIS-NDM-1 with masking approach and negative selections

Three selection rounds (SR11.2_Mask–14.2_Mask) were conducted with negative selections against unrelated mPD1-HIS or with unrelated mPD1-HIS and CFHR1-HIS prior to target incubation. Furthermore, HIS-NDM-1-coupled beads were pre-incubated with NDM1-H14-01-FR to mask the HIS-tag. Subsequently, enriched DNA-pools from SR 14.2_Mask and 14.2_Mask_Control were sequenced by NGS and analyzed bioinformatically. Enrichment was higher than for SR11.1 and 14.1, with 57.1% unique sequences for SR14.2_Mask. SR14.2_Mask_Control was also enriched, with 50.9% unique sequences. For SR14.2_Mask, the most enriched pattern accounted for 3.52% of total sequences. Six sequence families were identified, and 20 representatives were selected and synthesized for characterization of binding properties. The 20 representatives included members of each sequence family, as well as the most enriched clones on the condition that their percentage of total sequences was higher for SR14.2_Mask than for SR14.2_Mask_Control.

### Identification of NDM1-H14.2_43 as an NDM-1 specific aptamer

**Figure 4:**
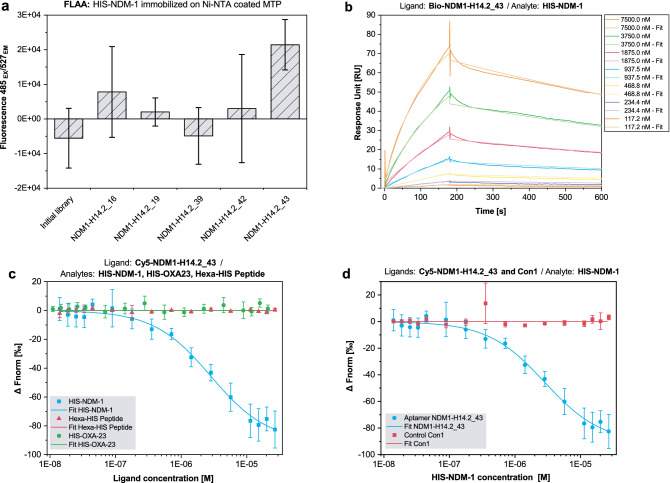
Characterization of NDM1-H14.2_43 binding to NDM-1 by FLAA, SPR and MST. HIS-NDM-1 was immobilized on a Ni-NTA-coated micro titer plate to reduce HIS-tag accessibility and incubated with promising aptamer candidates (**a**). Binding to NDM-1 was retained for the aptamer candidate NDM-1-H14.2_43. Immobilization of 5′-biotinylated NDM1-H14.2_43 on an SPR sensor chip and subsequent incubation with varying HIS-NDM-1 concentrations resulted in a K_D_ of 7.45 × 10^−7^ M (**b**). Cross-reactivity with HIS-OXA-23 and the hexa-HIS peptide, as well as non-specific binding of Con1, was tested using MST (**c**, **d**) whereby sequence-dependent, specific binding to NDM-1 could be confirmed. Error bars reflect the standard deviation of signals from three different target-coated wells plus the standard deviation from three negative controls wells in FLAA and the standard deviation from three different, independent experiments in MST.

As a first screening approach, aptamer candidates were tested for binding to HIS-NDM-1 and mPD1-HIS by FLAA using Greiner FLUOTRAC™ 600 microplates (Supplementary Fig. S2). Compared to SR14.1, binding signals were magnitudes lower. Hence, the combinatorial approach of negative selection with unrelated proteins and aptamer masking of the HIS-tag was sufficient to prevent prominent HIS-tag binding for all tested sequences. For sequences NDM1-H14.2_16, _19, _39, _42, and _43, binding signals were detected that were higher for HIS-NDM-1 than for mPD1-HIS, exceeded the signal of the initial library, and were therefore chosen for further characterization. To reduce HIS-tag accessibility, in FLAA HIS-NDM-1 was immobilized on a Ni-NTA-coated MTP via its HIS-tag and incubated with promising aptamer candidates (Fig. 4a). Here, a stable fluorescence signal, exceeding that of the initial library, used as a negative control, was detected for NDM1-H14.2_43. Therefore, this clone was chosen for quantitative characterization by MST and SPR (Fig. 4b–d). Specificity of the binding event was assessed using MST. 5′-Cy5 labeled NDM1-H14.2_43 was tested for binding to HIS-NDM-1, HIS-OXA-23, and the hexa-HIS peptide. In addition, an unrelated, 5′-Cy5-labelled DNA sequence (Con1) was used to evaluate non-specific DNA-binding to HIS-NDM-1. NDM1-H14.2_43, showed no binding to HIS-OXA-23 and hexa-HIS peptide, but to HIS-NDM-1 with a K_D_ of 2.82 × 10^−6^ M. The unrelated control aptamer Con1, did not bind to HIS-NDM-1. For SPR measurement, 5′-biotinylated NDM1-H14.2_43 was immobilized on an SPR sensor chip as a ligand and increasing concentrations of HIS-NDM-1 were injected as analytes. Binding parameters were calculated as K_D_ = 7.45 × 10^−7^ M, k_a_ = 1.00 × 10^3^ M^−1^ s^−1^, and k_d_ = 7.48 × 10^−4^ s^−1^. To further investigate sequence dependence of binding, 5′-biotinylated scramble control ConSc was used as a negative control aptamer in SPR (Supplementary Fig. S3). When compared to NDM1-H14.2_43, response units upon injection of HIS-NDM-1 were strongly decreased for ConSc. Kinetic parameters could not be determined as k_a_ was outside of the measurable range. Thus, sequence-dependent binding, specifically to HIS-NDM-1, could be validated. K_D_-values and kinetic parameters of the NDM-1 binding aptamer NDM1-H14.2_43 and controls are summarized in Table 2.

**Table 2:** Quantitative characterization of binding parameters of NDM1-H14.2_43 and control aptamers ConSc and Con1 using SPR and MST.

Method	Ligand	Analyte	k_a_ (1/Ms)	k_d_ (1/s)	K_D_ (M)	Chi^2^ (RU^2^)	K_D_ confidence (M)
SPR	Bio-NDM1-H14.2_43	HIS-NDM-1	1.00E+03	7.48E−04	7.45E−07	0.70	–
	Bio-ConSc		–	–	–	–	–
MST	Cy5-Con1	HIS-NDM-1	–	–	–	–	–
	Cy5-NDM1-H14.2_43	HIS-NDM-1			2.82E−06	–	3.77E−07
		HIS-OXA-23			–	–	–
		Hexa-HIS-peptide			–	–	–

## Discussion

Selection was performed against HIS-NDM-1, with negative selections against up to two other HIS-tagged carbapenemases. After eleven selection rounds, slight pool enrichment was detected, but selected aptamer candidates did not show binding to HIS-NDM-1. Negative selections against closely related molecules exert a strong selection pressure on the ssDNA pool^21^. Therefore, selection was progressed for three rounds without negative selections against other carbapenemases. After 14 SR, HIS-tag binding was indicated by SPR and confirmed by MST. The most promising aptamer candidate, NDM1-H14-01, and its truncated version, NDM1-H14-01-FR, were chosen for quantitative binding studies. Binding constants of both aptamers varied from the picomolar to the low nanomolar range for different proteins and different methods. Aptamers that bind to HIS-tags with high affinity have already been selected and published. Tsuji et al. published RNA aptamers that bind to polyhistidine, where the best binder, Shot47, had a K_D_ of 3.78 × 10^−12^ M when incubated with various concentrations of HIS-tagged macrophage migration inhibitory factor (HIS-MIF)^22^. In addition, Doyle et al. obtained a U.S. patent covering three DNA aptamers (6H1, 6H5, 6H7) characterized for binding to various HIS-tagged proteins that exhibited K_D_s of 8.00 × 10^−11^–1.76 × 10^−7^ M^23^. Like NDM1-H14-01 and NDM1-H14-01-FR, all published aptamers were characterized by SPR and the K_D_s are in a comparable range to those of our aptamers. However, as with NDM1-H14-01 and NDM1-H14-01-FR, the K_D_s of 6H1, 6H5, and 6H7 varied for different HIS-tagged proteins. Hence, for masking the HIS-tag of NDM-1, it seemed advisable to use the HIS-tag binding aptamer that was selected against and characterized for binding to HIS-NDM-1. For other HIS-tagged proteins, an evaluation of the binding properties of different, potential masking aptamers may be useful before starting a new aptamer selection. Variations in K_D_-values for different HIS-tagged proteins are well in line with differences in HIS-tag accessibility^24^. The discrepancy in K_D_-values calculated from SPR and MST may be explained by two factors. On the one hand, a synthetic hexa-HIS peptide was used for MST, whereas HIS-tagged proteins were chosen as analytes for SPR. Here, the protein scaffold surrounding the HIS-tag may have had a positive effect on aptamer binding. On the other hand, differences in the methods themselves may be explanatory. For SPR, the aptamer is immobilized on a chip and the analyte is free in solution, while both ligands and analytes are free in solution for MST. Furthermore, different labels were used. For SPR, the aptamer was 5′-biotinylated, while for MST, 5′-Cy5 labels were used. Despite the difference in K_D_ values obtained by the two methods, highly affine binding of the NDM1-H14-01 aptamer and its truncated version to HIS-tags was shown.

Since selection was initially aimed at enriching binders against NDM-1 rather than polyhistidine tags, a new selection strategy was designed, in which NDM1-H14-01-FR was used to mask the N-terminal HIS-tag of NDM-1. Another possible approach to avoid selection of HIS-tag binding aptamers is tag removal prior to selection. Proteases such as enterokinase, thrombin, and factor Xa can be used to remove affinity tags via specific cleavage sites that can be inserted between the protein of interest and its respective affinity tag^25^. However, unwanted, non-specific cleavage sites can compromise protein integrity. Viral proteases such as human rhinovirus 3C protease (3CP or PreScission) and tobacco etch virus (TEV) protease are known to be more specific. However, some fusion proteins are inherently poor substrates for the TEV protease, which poses the risk of incomplete cleavage^26^. In addition, the need for purification after cleavage can lead to a reduction in yield. If the desired protein is commercially available, cleavage sites may not be included, and custom synthesis may increase acquisition costs. Therefore, tag masking, for some proteins of interest, may be a more appropriate approach to avoid selecting aptamers that bind to tags. Masking of dominant epitopes in proteins with existing aptamers is known in the aptamer community but has, to the best of our knowledge, not been applied to HIS-tags yet^27^. In this study, we demonstrate the selection of an NDM-1-specific aptamer by masking the HIS-tag of the target molecule. The applicability of our approach to other affinity tags would need further investigation. In particular, masking might be difficult for tags with higher molecular size, such as glutathione S-transferase tag or F_c_-tag, because high molecular weight tags may have multiple aptamer binding sites.

After three rounds of selection with the masking approach in combination with negative selections against one or two unrelated HIS-tagged proteins, the aptamer candidate NDM1-H14.2_43 was identified to specifically bind to HIS-NDM-1 but not to HIS-OXA-23, nor to the hexa-HIS peptide. The dissociation constant, calculated for non-HIS mediated NDM-1 binding, was in the moderate range and may allow detection of this prevalent and health-hazardous carbapenemase. Here, SPR-based K_D_ calculation resulted in higher affinity values than MST-based K_D_ calculation. Thus, immobilization of the aptamer may be expedient for future detection assay formats. To date, detection of NDM-1 can be done mainly by genotypic, phenotypic, or lateral flow assays. Genotype-based tests such as PCR are highly sensitive and accurate, but require well-trained personnel and usually cannot detect mutant genes. Phenotypic assays detect the in vitro enzyme activity of carbapenemases, not necessarily distinguishing between different carbapenemase types. Detection of carbapenemases, which also allows discrimination between different enzyme types and does not require trained technicians, can be accomplished with antibody-based lateral flow devices (LFD) such as the NG-Test® CARBA 5^28^. Antibody-based LFDs are valuable tools for simple and effective point-of-care detection of carbapenemases. However, aptamer-based LFDs have certain advantages over antibody-based systems. Their long shelf life and stability, as well as chemical synthesis that allows for easy chemical modification and enables low-cost production with extremely low batch-to-batch variability, make aptamers ideal candidates for implementation in LFDs^29^. Therefore, NDM1-H14.2_43 may serve as a valuable detection probe when used in a competitive LFD assay format. In addition, an aptamer-antibody sandwich assay format can be developed if the aptamer and antibody have different binding sites. Sandwich assay formats are known for their high specificity and sensitivity^30^.

In conclusion, we presented a strategy to prevent and reverse selection of HIS-tag binding sequences during aptamer selection. We further introduce an aptamer with specific binding properties to the highly prevalent carbapenemase NDM-1 and its potential for future use as a detection probe. Carbapenem resistance in Enterobacteriaceae poses a worldwide health risk. Specific detection of resistance markers is a prerequisite for containing transmission and to facilitate appropriate treatment. Our NDM1-H14.2_43 aptamer may be used for the development of specific, cost efficient, and easy-to-use detection methods and thereby prevent unnecessary spread of carbapenem resistant bacteria and to help avoid medical malpractice.

## Methods

### Target preparation

N-terminally hexa-HIS-tagged carbapenemases NDM-1 (derived from *E. coli*), OXA-23 (derived from *A. baumannii*), and KPC-2 (derived from *K. pneumonia*) were purchased from Hölzel Diagnostica Handels GmbH (Köln, Germany). HIS-NDM-1 and HIS-OXA-23 were supplied as a filtered solutions in PBS, while HIS-KPC-2 was supplied as filtered solution in 100 mM NaH_2_PO_4_, 0.3 M NaCl, and 4 M Urea. For negative selections with unrelated proteins, C-terminally HIS-tagged mPD1 and CFHR1 were purchased from Abcam plc (Cambridge, US) and Hölzel Diagnostica Handels GmbH (Köln, Germany). The hexa-HIS peptide was purchased from BIOTREND Chemikalien GmbH (Köln, Germany). For selection, 10 µg of all proteins were covalently coupled to 1 mg of magnetic beads (Dynabeads™ M-280 Tosylactivated, Thermo Fisher Scientific Inc, Waltham, USA) for 16 h at 37 °C using a rotator. Coupling efficiency was determined by protein quantification in the supernatant prior to and after bead incubation using the Micro BCA™ Protein-Assay-Kit (Thermo Fisher Scientific Inc, Waltham, USA).

### Nucleic acid preparation

The initial library (DSB-44) was composed of a 44 nt randomized sequence, flanked by two 18 nt constant primer regions (GTATCTGGTGGTCTATGG–N(44)–GCATAGACGACGAAGAAC) and purchased from Ella Biotech GmbH (Fürstenfeldbruck, Germany). Primers were purchased from TIB Molbiol Syntheselabor GmbH (Berlin, Germany) and aptamers including modifications from biomers.net GmbH (Ulm, Germany) and Integrated DNA Technologies Inc (Coralville, USA). Oligonucleotides were synthesized by standard solid-phase DNA synthesis and purified either by HPLC or standard desalting. Sequences of nucleic acids used in this work can be found in Supplementary Table S2.

### Semiautomatic in vitro selection

Aptamers were selected using a semiautomatic selection procedure as previously described^31,32^. Prior to every selection round, ssDNA pools were activated by denaturation at 92 °C for 3 min followed by slowly cooling down to room temperature (RT) for approximately 30 min (refolding step) in binding buffer BP-T (20 mM Tris–HCl [pH 7.4], 140 mM NaCl, 5 mM MgCl_2_, 1 mM CaCl_2_, 1 mM KCl, 0.1% BSA, and 0.05% [v/v] Tween® 20). Subsequently, ssDNA libraries and enriched ssDNA pools were incubated with uncoupled tosylactivated magnetic beads as a negative selection step to reduce matrix binders. Negative selection steps with other HIS-tagged carbapenemases or the unrelated, HIS-tagged proteins mPD1-HIS and CFHR1-HIS were introduced and varied over the course of selection. For SR 11.2_Mask–14.2_Mask, HIS-NDM-1-coated beads were pre-incubated with HIS-tag binding aptamer NDM1-H14-01-FR for 1 h in BP-T at RT using a rotator (for further information on negative selections and the masking approach, see section below and Supplementary Table S1). As a selection step, ssDNA pools were either incubated with the HIS-NDM-1-coated beads (target selection) or tosylactivated magnetic beads (control selection) for 1 h at RT in BP-T. Non-binding sequences were removed by extensive washing steps in BP-T. Washing was performed for 3–30 min in volumes up to 2.5 × incubation volume. Remaining sequences were eluted with 8 M urea in BP-T for 30 min at 65 °C and 900 rpm using a thermo shaker and precipitated with 2-propanol at − 20 °C overnight. Eluted ssDNA was amplified by PCR with phosphorylated reverse primers and analyzed by gel electrophoresis. Subsequent purification of the single stranded forward (aptamer) strand was enabled by lambda exonuclease digestion (1.5 U/µg dsDNA)^33^.

### Negative selections and masking approach

From SR1 onwards, pre-incubation with varying amounts of tosylactivated beads was used as a negative selection step to reduce matrix binding sequences (Supplementary Table S1). Further negative selections were introduced for SR5.1–11.1 (pre-incubation with HIS-KPC-2-coated beads) and SR8.1–11.1 (pre-incubation with HIS-OXA-23-coated beads). Negative selection against carbapenemase-coated beads was stopped for SR11.1–14.1. Here, only uncoated magnetic beads were used to reduce matrix binders. After SR14.1, the selection strategy was changed and SR11.2_Mask–14.2_Mask were conducted with negative selections against tosylactivated beads and mPD1-HIS-coated beads. For SR13.2_Mask and SR14.2_Mask, negative selection was additionally performed against CFHR1-HIS-coated beads. Moreover, for SR11.2_Mask–14.2_Mask, prior to the selection step, HIS-NDM-1-coated beads were pre-incubated with the HIS-tag binding sequence NDM1-H14.1-01-FR to mask the HIS-tag. Detailed information on negative selection and masking of the HIS-tag can be found in Supplementary Table S1.

### Next generation sequencing and bioinformatic analysis of enriched ssDNA pools

Enriched pools were sequenced after selection rounds 10.1, 11.1, 11.1_Control, 13.1, 14.1, 14.1_Control, as well as 14.2_Mask and 14.2_Mask_Control. Enriched ssDNA pools were prepared for sequencing using the TruSeq® Nano DNA Library Prep Kit (Illumina, Inc, San Diego, USA). Sequencing was performed using the Mini Seq Mid Output Kit on a Miniseq sequencing platform (Illumina, Inc, San Diego, USA). Paired end mode was chosen with 150 reads per direction and data was bioinformatically analyzed using AptaAnalyzer™-SELEX (AptaIT GmbH, Martinsried, Germany).

### Fluorescence dye-linked aptamer assay (FLAA)

As a first screening of potential aptamers, FLAAs were performed as previously described^34^ using an EnVision® 2105 multimode plate reader (PerkinElmer Inc., Waltham, USA). All reagents were diluted to their final concentrations in BP-T. Targets were immobilized either on Greiner FLUOTRAC™ 600 microplates (Merck KGaA, Darmstadt, Germany) for three hours at RT and 300 rpm followed by 14 h at 18 °C, or on Pierce™ Nickel Coated Plates (Thermo Fisher Scientific, Waltham, USA) for one hour at RT and 300 rpm. Wells were incubated with either 0.6 µg protein in PBS or with PBS only (negative control). Subsequently, wells were washed thrice with BP-T and incubated with 60–100 pmol of activated ssDNA for one hour at RT and 300 rpm. Micro titer plates (MTP) were washed thrice with BP-T and incubated with a 1:200 dilution of Quant-iT™ OliGreen™ ssDNA reagent (Thermo Fisher Scientific, Waltham, USA) for up to 20 min. Fluorescence was measured thrice (excitation 485 nm, emission 527 nm) after 12 min of dye incubation for clones from SR11.1 and 14.1 and after 20 min of dye incubation for clones from SR14.2_Mask. Measurements were performed in duplicate or triplicate. Fluorescence values were averaged, and negative control values were subtracted.

### Surface plasmon resonance spectroscopy (SPR)

SPR was performed using a BIAcore T200 facility (GE healthcare, Chicago, USA). The system was operating at 25 °C at flow rates of 10–30 µl/min. BP-T* (20 mM Tris–HCl [pH 7.4], 140 mM NaCl, 5 mM MgCl_2_, 1 mM CaCl_2_, 1 mM KCl, and 0.005% [v/v] Tween® 20) was used as the running buffer. HC200M sensor chips (XanTec bioanalytics GmbH, Düsseldorf, Germany) were primed twice with ddH_2_O and activated with 0.2 M EDC and 0.1 M NHS for 180 s. Regenerations were performed with 20 mM Na_2_CO_3_.

For aptamer candidate screenings, protein ligands were immobilized via amine coupling for 180 s and the chip was blocked with 1 M ethanolamine for 420 s at a flow rate of 20 µl/min. Aptamer candidates were activated, diluted to 1 µM and injected with 180 s association and 300 s dissociation at a flow rate of 30 µl/min.

For kinetic measurements, 20 µg/ml NeutrAvidin was immobilized via amine coupling for 120 s and the chip was blocked with 1 M ethanolamine for 420 s at a flow rate of 20 µl/min. 5′-biotinylated aptamers were activated, diluted to 0.1 µM in BP-T*, and immobilized at a flow rate of 10 µl/min to approximately 100 RU. NeutrAvidin blocking was performed with 40 µM biotin for 120 s at a flow rate of 20 µl/min. Analytes were diluted in BP-T* and injected to both the ligand-bound and the reference channel with 180 s association and 600 s dissociation at a flow rate of 30 µl/min. Kinetic parameters were calculated using BIAevaluation software (version 3.1, BIAcore). SPR scramble control aptamer ConSc was designed using The Sequence Manipulation Suite®^35^.

### MicroScale Thermophoresis (MST)

MST experiments were conducted using constant concentrations of the 5′-Cy5-labelled aptamers NDM1-H14-01, NDM1-H14.2_43, and Con1, and varying concentrations of HIS-NDM-1, HIS-OXA-23, and the hexa-HIS peptide. For characterization of NDM1-H14-01 binding to the hexa-HIS peptide, NDM1-H14-01 and Con1 were diluted to 20 nM in BP-T. A serial dilution of the hexa-HIS peptide (8960–2.72 nM) was prepared in BP-T and 10 µl of each aptamer and target solution were mixed. For characterization of selective binding of NDM1-H14.2_43 to HIS-NDM-1, aptamers NDM1-H14.2_43 and Con1 were diluted to 42 nM in BP-T and activated as described above. Serial dilutions of HIS-NDM-1, the hexa-HIS peptide (32.3–0.017 µM), and HIS-OXA-23 (25.3–0.013 µM) were prepared in BP-T, and 1 µl of each aptamer was mixed with 5 µl of ligand solution. Binding experiments were performed on a Monolith NT.115 (NanoTemper Technologies, Munich, Germany) with 40–70% excitation power and 40–60% MST power using standard capillaries. The recorded fluorescence was normalised to ΔF_norm_ in per mill and fitted utilizing the K_D_ formula derived from the law of mass action by MO.*Affinity Analysis* software as previously described^36^. All measurements were performed in triplicate.

## Supplementary Information

Supplementary Information.

## Data Availability

All datasets generated during and/or analyzed during the current study are available from the corresponding author. Next generation sequencing data is available in the Sequence Read Archive (SRA) repository, Accession number PRJNA830749.
