# Supplementary material for: The use of high-affinity polyhistidine binders as masking probes for the selection of an NDM-1 specific aptamer
Source: Sci Rep. 2022 May 13;12:7936. doi: 10.1038/s41598-022-12062-2 (PMC9106695; doi:10.1038/s41598-022-12062-2)
Supplement: Supplementary file 1 — Supplementary Information. [file 41598_2022_12062_MOESM1_ESM.pdf]

Supplementary Figures and Tables

The use of high-affinity polyhistidine binders as masking probes for the selection of an NDM-1 specific aptamer

Wiebke Sabrowski [1, 2], Nico Dreymann [1, 3], Anja Möller [1], Denise Czepluch [1], Patricia P. Albani [4], Dimitrios Theodoridis [4] and Marcus M. Menger [1*]

[1] Fraunhofer Institute for Cell Therapy and Immunology, Branch Bioanalytics and Bioprocesses (IZI-BB), Am Mühlenberg 13, 14476 Potsdam, Germany

[2] Institute of Chemistry and Biochemistry – Biochemistry, Freie Universität Berlin, Takustr. 6, 14195, Berlin, Germany

[3] Institute of Biochemistry and Biology, University of Potsdam, Karl-Liebknecht Strasse 24-25, 14476, Potsdam-Golm, Germany

[4] nal von minden GmbH, Robert-Bosch-Breite 23, 37079 Göttingen, Germany

*marcus.menger@IZI-BB.fraunhofer.de

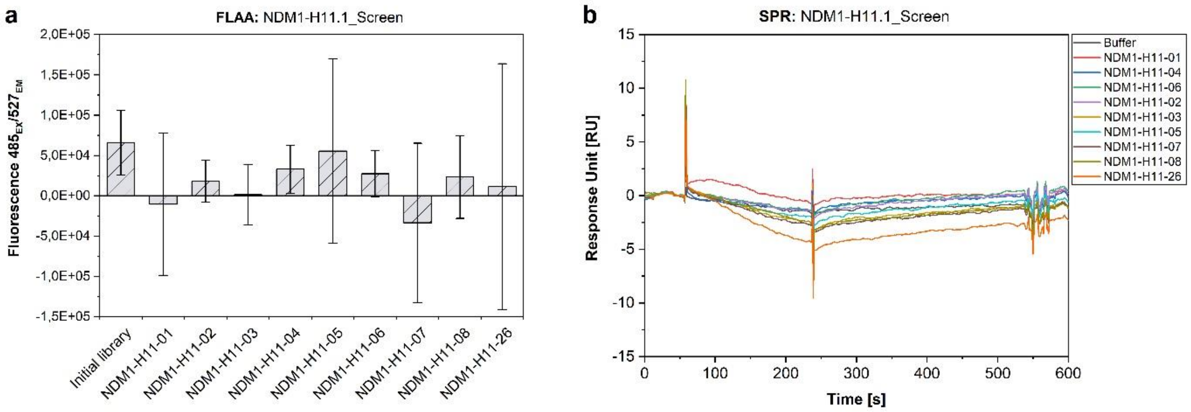

Supplementary Figure S1: FLAA and SPR Screen after SR11.1. HIS-NDM-1 was immobilized on either a FLUOTRAC™ 600 microplate and incubated with initial library and aptamer candidates (a) or on a SPR sensor chip and aptamer candidates were injected as analytes (b). No binding was detected. Error bars reflect the standard deviation of signals from three target-coated wells plus the standard deviation from three negative controll wells in FLAA.

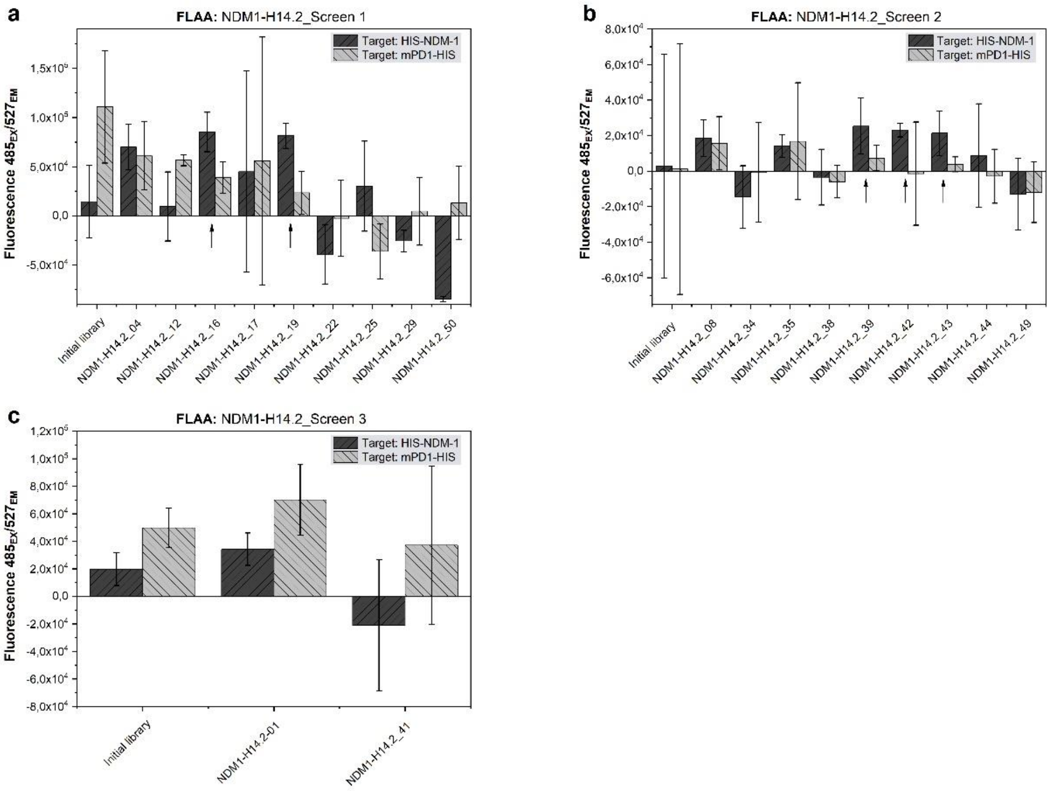

Supplementary Figure S2: FLAA screening after SR14.2. HIS-NDM-1 and mPD1-HIS were immobilized on a FLUOTRAC™ 600 microplate and incubated with initial library and aptamer candidates. Aptamer candidates for which binding signals for HIS-NDM-1 exceeded that for mPD1-HIS were chosen for further characterization. Error bars reflect the standard deviation of signals from three target-coated wells plus the standard deviation from three negative controll wells in FLAA.

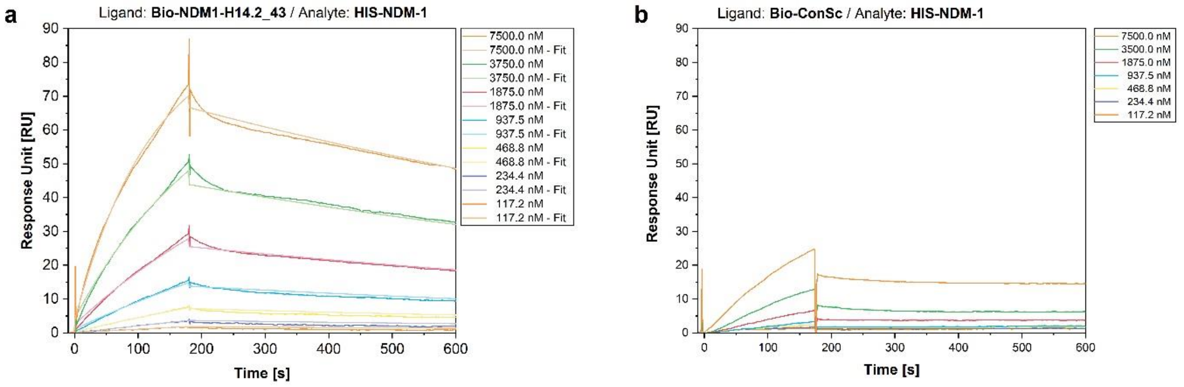

Supplementary Figure S3: SPR measurement for ConSc (b) in comparison to NDM1-H14.2_43 (a). Response units upon injection of increasing concentrations of HIS-NDM-1 were strongly reduced for ConSc. Kinetic parameters could not be determined.

Supplementary Table S1: Selection against HIS-NDM-1: Negative selections and masking approach.

<table>
<tr><th>SR</th><th>ssDNA [pmol]</th><th>NDM-1-coated beads [pmol]</th><th>NDM-1-coated beads [µl]</th><th>Beads only [µl]</th><th>KPC-2-coated beads [pmol]</th><th>OXA-23-coated beads [pmol]</th><th>mPD1-coated beads [pmol]</th><th>CFHR1-coated beads [pmol]</th><th>NDM1-H14-01-FR (masking) [pmol]</th></tr>
<tr><td>1.1</td><td>1000</td><td>100</td><td>13.2</td><td>1x 13.2</td><td rowspan="6">-</td><td rowspan="15">-</td><td rowspan="28">-</td><td rowspan="31">-</td><td rowspan="28">-</td></tr>
<tr><td>1.2</td><td>1000</td><td>100</td><td>13.2</td><td>1x 13.2</td></tr>
<tr><td>2.1</td><td>200</td><td>40</td><td>5.6</td><td>2x 5.6</td></tr>
<tr><td>3.1</td><td>200</td><td>40</td><td>5.3</td><td>2x 5.3 + 1x 10.6</td></tr>
<tr><td>3.1_ Control</td><td>200</td><td>-</td><td>-</td><td>2x 5.3 + 1x 10.6, selection step with 5.3</td></tr>
<tr><td>4.1</td><td>120</td><td>40</td><td>5.3</td><td>2x 5.3 + 1x 10.6</td></tr>
<tr><td>5.1</td><td>120</td><td>40</td><td>3.8</td><td>1x 3.8 + 1x 7.6</td><td>40</td></tr>
<tr><td>5.1_ Control</td><td>120</td><td>-</td><td>-</td><td>1x 3.8 + 1x 7.6, selection step with with 3.8</td><td>40</td></tr>
<tr><td>6.1</td><td>120</td><td>40</td><td>3.8</td><td>1x 3.8 + 1x 7.6</td><td>40</td></tr>
<tr><td>6.1_ Control</td><td>120</td><td>-</td><td>-</td><td>1x 3.8 + 1x 7.6, selection step with 3.8</td><td>40</td></tr>
<tr><td>7.1</td><td>120</td><td>40</td><td>3.8</td><td>1x 3.8 + 1x 7.6</td><td>40</td></tr>
<tr><td>6.2</td><td>120</td><td>40</td><td>5.1</td><td>1x 5.1 + 1x 10.2</td><td>40</td></tr>
<tr><td>6.2_ Control</td><td>120</td><td>-</td><td>-</td><td>1x 5.1 + 1x 10.2, selection step with 5.3</td><td>40</td></tr>
<tr><td>7.2</td><td>120</td><td>40</td><td>5.1</td><td>1x 5.1 + 1x 10.2</td><td>40</td></tr>
<tr><td>7.2_ Control</td><td>120</td><td>-</td><td>-</td><td>1x 5.1 + 1x 10.2, selection step with 5.3</td><td>40</td></tr>
<tr><td>8.1</td><td>80</td><td>40</td><td>5.1</td><td>1x 5.1 + 1x 10.2 + 1x 15.3</td><td>40</td><td>40</td></tr>
<tr><td>8.1_ Control</td><td>80</td><td>-</td><td>-</td><td>1x 5.1 + 1x 10.2 + 1x 15.3, selection step with 5.3</td><td>40</td><td>40</td></tr>
<tr><td>9.1</td><td>80</td><td>40</td><td>3.4</td><td>1x 3.4 + 1x 6.8 + 1x 10.2</td><td>40</td><td>40</td></tr>
<tr><td>10.1</td><td>80</td><td>40</td><td>8.5</td><td>1x 8.5 + 1x 17 + 1x 25.5</td><td>80</td><td>80</td></tr>
<tr><td>10.1_ Control</td><td>80</td><td>40</td><td>-</td><td>1x 8.5 + 1x 17 + 1x 25.5, selection step with 8.5</td><td>80</td><td>80</td></tr>
<tr><td>11.1</td><td>80</td><td>40</td><td>3.4</td><td>1x 3.4 + 1x 6.8 + 1x 10.2, selection step with 3.4</td><td>80</td><td>80</td></tr>
<tr><td>11.1_ Control</td><td>80</td><td>40</td><td>-</td><td>1x 3.4 + 1x 6.8 + 1x 10.2, selection step with 3.4</td><td>80</td><td>80</td></tr>
<tr><td>12.1</td><td>80</td><td>40</td><td>3.8</td><td>1x 3.8 + 1x 7.6 + 1x 11.4</td><td rowspan="12">-</td><td rowspan="12">-</td></tr>
<tr><td>12.1_ Control</td><td>80</td><td>-</td><td>-</td><td>1x 3.8 + 1x 7.6 + 1x 11.4, selection step with 3.8</td></tr>
<tr><td>13.1</td><td>80</td><td>40</td><td>5.3</td><td>1x 15.9 + 1x 10.6 + 1x 5.3</td></tr>
<tr><td>13.1_ Control</td><td>80</td><td>-</td><td>-</td><td>1x 15.9 + 1x 10.6 + 1x 5.3, selection step with 5.3</td></tr>
<tr><td>14.1</td><td>80</td><td>40</td><td>5.3</td><td>1x 15.9 + 1x 10.6 + 1x 5.3</td></tr>
<tr><td>14.1_ Control</td><td>80</td><td>40</td><td>-</td><td>1x 15.9 + 1x 10.6 + 1x 5.3, selection step with 5.3</td></tr>
<tr><td>11.2_Mask</td><td>80</td><td>40</td><td>4.0</td><td>1x 8 + 1x 4</td><td>40</td><td>400</td></tr>
<tr><td>12.2_Mask</td><td>80</td><td>40</td><td>4.0</td><td>1x 8 + 1x 4</td><td>80</td><td>400</td></tr>
<tr><td>12.2_Mask_ Control</td><td>80</td><td>-</td><td>-</td><td>1x 8 + 1x 4, selection step with 4</td><td>80</td><td>400</td></tr>
<tr><td>13.2_Mask</td><td>80</td><td>40</td><td>4.0</td><td>1x 8 + 1x 4</td><td>40</td><td>40</td><td>400</td></tr>
<tr><td>14.2_Mask</td><td>80</td><td>20</td><td>2.0</td><td>1x 4 + 1x 2</td><td>40</td><td>20</td><td>200</td></tr>
<tr><td>14.2_Mask_ Control</td><td>80</td><td>-</td><td>-</td><td>1x 4 + 1x 2, selection step with 2</td><td>40</td><td>20</td><td>200</td></tr>
</table>

Supplementary Table S2: Aptamer candidate sequences used in this work.

NDM1-H11-01	GTATCTGGTGGTCTATGGCCCGGGTCAACCGTTCTTCTCGTTTCCGTTATTTTGTCTTCTATGTTCTTCGTCGTCTATGC
NDM1-H11-02	GTATCTGGTGGTCTATGGCATAGACGACGAAGAACAGGACATTTTTGGTTTTTGGGTACTCCTCCGTTCTTCGTCGTCTATGC
NDM1-H11-03	GTATCTGGTGGTCTATGGCGGAGGTGTTCTGAGTAGTCAGTTCAAGTCGCTTAATTTTCGGGGTTCTTCGTCGTCTATGC
NDM1-H11-04	GTATCTGGTGGTCTATGGCACAGTGAAAATTCTTCGTCCCAATCTCTCTCTTCGACTCATATGTTCTTCGTCGTCTATGC
NDM1-H11-05	GTATCTGGTGGTCTATGGGTCGTCTCTTTTTTGTTGTTTTTTTTTGGTTTCCCGTGTTCTTCGTCGTCTATGC
NDM1-H11-06	GTATCTGGTGGTCTATGGCCCCGGAACCTTCGTCGATAGTTCTTCTCAACAGATACATTCCGGTTCTTCGTCGTCTATGC
NDM1-H11-07	GTATCTGGTGGTCTATGGCGGGACGTCGTCTAACGGTTCTTCTCTTGTCCTTTTTTGTGTGTGTTCTTCGTCGTCTATGC
NDM1-H11-08	GTATCTGGTGGTCTATGGCGGCGATAACAAAAAAAAAAACAAATTTGCTGTATCTGGTGGTCTATGGTTCTTCGTCGTCTATGC
NDM1-H11-26	GTATCTGGTGGTCTATGGCGAGGGGTGAGCTAAAAAAAAAAAAAAAATGTCCACGTCGTGGGGGGTTCTTCGTCGTCTATGC
NDM1-H14-01	GTATCTGGTGGTCTATGGCGTCATAGGTACCATGGGGGACTGCTCGGGATTGCGGATTCATGGCATAGACGACGAAGAAC
NDM1-H14-01-FR	CGTCATAGGTACCATGGGGGACTGCTCGGGATTGCGGATTCATG
NDM1-H14-02	GTATCTGGTGGTCTATGGCCAGTATGGCTGTTCAAGTCTTCAGAGGGTCTGCTCGGGATTGCGCATAGACGACGAAGAAC
NDM1-H14-03	GTATCTGGTGGTCTATGGCATAGACGACGAAGAACAGGACATTTTTGGTTTTTGGGTACTCCTCCGCATAGACGACGAAGAAC
NDM1-H14-04	GTATCTGGTGGTCTATGGCATAGACGACGAAGAACAGCCGCAAAAAATAAAAGCCTAACGAGGGGCATAGACGACGAAGAAC
NDM1-H14-05	GTATCTGGTGGTCTATGGCCCCGGAACCTTCGTCGATAGTTCTTCTCAACAGATACATTCCGGCATAGACGACGAAGAAC
NDM1-H14-06	GTATCTGGTGGTCTATGGCATAGACGACGAAGAACAACAAAAAGAGCGAACAAATCGGGGCTGGTGGGCATAGACGACGAAGAAC
NDM1-H14-07	GTATCTGGTGGTCTATGGCATAGACGACGAAGAACAGGCGGGCATAGACGACGAAGAACAGGCGGGCATAGACGACGAAGAAC
NDM1-H14-09	GTATCTGGTGGTCTATGGCATAGACGACGAAGAACACTAACACGTTTTTTTACCTTATACTTGCGGGCATAGACGACGAAGAAC
NDM1-H14-10	GTATCTGGTGGTCTATGGCGGCGATAACAAAAAAAAAACAAATTTGCTGTATCTGGTGGTCTATGGCATAGACGACGAAGAAC
NDM1-H14-12	GTATCTGGTGGTCTATGGCATAGACGACGAAGAACAGTGTATCTGGTGGTCTATGGCATAGACGACGAAGAAC
NDM1-H14-17	GTATCTGGTGGTCTATGGCGGAGGTGTTCTGAGTAGTCAGTTCAAGTCGCTTAATTTTCGGGGCATAGACGACGAAGAAC
NDM1-H14-18	GTATCTGGTGGTCTATGG CCCGGGTCAACCGTTCTTCTCGTTTCCGTTATTTTGTCTTCTATGCATAGACGACGAAGAAC
NDM1-H14-25	GTATCTGGTGGTCTATGGCAAACGTTCTGAGTTAGGCTTATTTTTCTCTCTTCCCTTCGCCGCATAGACGACGAAGAAC
NDM1-H14-39	GTATCTGGTGGTCTATGGCATAGACGACGAAGAACAATAAGGGGCAAATCTATTTAATTGGGGAGAGCATAGACGACGAAGAAC
NDM1-H14.2_04	GTATCTGGTGGTCTATGGCATAGACGACGAAGAACAATGAAACCCAATCCCTCTCCCCTTCGCCGCATAGACGACGAAGAAC
NDM1-H14.2_08	GTATCTGGTGGTCTATGGCCCCGGAACCTTCGTCGATAGTTCTTCTCAACAGATACATTCCGGCATAGACGACGAAGAAC
NDM1-H14.2_12	GTATCTGGTGGTCTATGGCCCCGTACCTATGACGCGCTTTTCTTTTTCTTATCTTTCCTTGTGCATAGACGACGAAGAAC
NDM1-H14.2_16	GTATCTGGTGGTCTATGGCCAACGAGAATCGGCAATCCTTAAACTGTGTATCTGGTGGTCTATGGCATAGACGACGAAGAAC

NDM1-H14.2_17	GTATCTGGTGGTCTATGGCATAGACGACGAAGAACAACCTTGAATCCAATTATCCCCAGCATAGACGACGAAGAAC
NDM1-H14.2_19	GTATCTGGTGGTCTATGGCCAAAATCCATCAATCCCGTTTTCATGTGCTCCGCTTACTCTTCGCATAGACGACGAAGAAC
NDM1-H14.2_22	GTATCTGGTGGTCTATGGCATAGACGACGAAGAACAGACCACTTATTTAATCCCACCCAGGCATAGACGACGAAGAAC
NDM1-H14.2_25	GTATCTGGTGGTCTATGGCCAGACTACTTTCAAAAACCAGACCATTTCATTTTAGTAAATCCGCATAGACGACGAAGAAC
NDM1-H14.2_29	GTATCTGGTGGTCTATGGCCAACAAATACAATTAACATGAAGCCGCCCTGTTTTTTCCTTCCGCATAGACGACGAAGAAC
NDM1-H14.2_34	GTATCTGGTGGTCTATGGCCAAGAATCCATCATATCTCCAATCCCAAGCTTTTATTTACTCAGCATAGACGACGAAGAAC
NDM1-H14.2_35	GTATCTGGTGGTCTATGGCCACAACATGAATCCGTTTCTAATTCCTTTTACCTTTTCTCTCAGCATAGACGACGAAGAAC
NDM1-H14.2_38	GTATCTGGTGGTCTATGGCAAGGGCGTTCTTCATCGGTTCTTCTCACATTTTCCTCCCACCAGCATAGACGACGAAGAAC
NDM1-H14.2_39	GTATCTGGTGGTCTATGGCGGAGGTCTAAAAAAAAAAAAACGCAATCCTATCTCATAATCCGCATAGACGACGAAGAAC
NDM1-H14.2_41	GTATCTGGTGGTCTATGGCGCTAGTTCTTCGCATCATTCCGCTTCCTTTCTTCTCCTTCTCCGCATAGACGACGAAGAAC
NDM1-H14.2_42	GTATCTGGTGGTCTATGGCATAGACGACGAAGAACACTACCCGCAAATTTTCACTGTAATCCGCATAGACGACGAAGAAC
NDM1-H14.2_43	GTATCTGGTGGTCTATGGCCGCTATCCGCAATCTGTCTCTTTTCTTTATTCTTTTACTCACCGCATAGACGACGAAGAAC
NDM1-H14.2_44	GTATCTGGTGGTCTATGGCATAGACGACGAAGAACATGAATGATCAACTAAGGGATAGCAATCTCGGCATAGACGACGAAGAAC
NDM1-H14.2_49	GTATCTGGTGGTCTATGGGCCCGGACTAAAAATCAAAATCATATCGACCGCAATCCCTTTCCGCATAGACGACGAAGAAC
NDM1-H14.2_50	GTATCTGGTGGTCTATGGCGCATCCCTATCCCGTCTCTTTTTGTATTTTTATTTTTGTTTCCGCATAGACGACGAAGAAC
Bio-NDM1-H14-01-FR	/5BiotinTEG/CGTCATAGGTACCATGGGGGACTGCTCGGGATTGCGGATTCATG
Bio-NDM1-H14-01	/5BiotinTEG/GTATCTGGTGGTCTATGGCGTCATAGGTACCATGGGGGACTGCTCGGGATTGCGGATTCATGGCATAGACGACGAAGAAC
Cy5-NDM1-H14-01	/5Cy5/GTATCTGGTGGTCTATGGCGTCATAGGTACCATGGGGGACTGCTCGGGATTGCGGATTCATGGCATAGACGACGAAGAAC
Bio-NDM1-H14.2_43	/5BiotinTEG/GTATCTGGTGGTCTATGGCCGCTATCCGCAATCTGTCTCTTTTCTTTATTCTTTTACTCACCGCATAGACGACGAAGAAC
Cy5-NDM1-H14.2_43	/5Cy5/GTATCTGGTGGTCTATGGCCGCTATCCGCAATCTGTCTCTTTTCTTTATTCTTTTACTCACCGCATAGACGACGAAGAAC
Cy5-Con1	/5Cy5/GGGAATTCGAGCTCGGTACCGGCTGCTTTGCTGCAGATTTGTGGGTGGGTGGGTGGTGATCTGCAGGCATGCAAGCTTGG
Bio-ConSc	/5BiotinTEG/TTATAACCGGTTATACTGGTTGTTCCTAGATGCTTCATGATGCCCAACCTTTGTCCCTGGCCCTGACATGTTTCGACTAA
